# Serum GPVI for Diagnostic Assessment and Short-Term Risk Stratification in Patients with Suspected Transient Ischemic Attack: A Prospective Observational Cohort Study

**DOI:** 10.3390/brainsci16060596

**Published:** 2026-05-30

**Authors:** Ömer Gençer, Mustafa Burak Sayhan, Mehmet Tahir Gokdemir, Eray Çeliktürk, Satuk Buğra Han Bozatlı, Rıza Serttaş

**Affiliations:** 1 Department of Emergency Medicine, Medicine Faculty, Trakya University, Edirne 22030, Turkey; omergencer3@gmail.com (Ö.G.); eraycelikturk@trakya.edu.tr (E.Ç.); 2 Department of Emergency Medicine, Medicine Faculty, Mardin Artuklu University, Mardin 47000, Turkey; tahirgokdemir@artuklu.edu.tr; 3 Emergency Department, Edirne Sultan 1st Murat State Hospital, Edirne 22030, Turkey; satbuhan@gmail.com; 4 Department of Medical Biology, Medicine Faculty, Trakya University, Edirne 22030, Turkey; serttasriza@outlook.com

**Keywords:** transient ischemic attack, glycoprotein VI, GPVI, platelet activation, ABCD2 score, PREDISC, stroke risk, emergency department, cerebrovascular events, biomarker

## Abstract

**Highlights:**

**What are the main findings?**

- Serum GPVI levels increased stepwise with the clinical likelihood of transient ischemic attack, showing the highest values in patients classified as “very likely TIA” by PREDISC.
- Higher serum GPVI levels were associated with ABCD2 scores ≥4 and with early cerebrovascular events within 2 and 7 days after emergency department presentation.

**What are the implications of the main findings?**

- Serum GPVI may provide a preliminary biological signal warranting further investigation to support clinical diagnosis in patients presenting with transient focal neurological symptoms.
- Combining GPVI measurement with established clinical scores may represent a hypothesis-generating basis for future studies aiming to improve short-term risk stratification and identify patients who require closer follow-up or intensified secondary prevention.

**Abstract:**

**Background/Objectives:** Transient ischemic attack (TIA) is a neurological emergency associated with a substantial early risk of ischemic stroke, yet its diagnosis remains clinically challenging because objective biomarkers are limited. Glycoprotein VI (GPVI), a platelet collagen receptor involved in platelet activation and thrombus formation, may reflect prothrombotic activity in cerebrovascular ischemia. This study aimed to evaluate the diagnostic assessment and short-term risk stratification value of serum GPVI levels in patients presenting to the emergency department with suspected TIA. **Methods:** This prospective observational cohort study included 85 adult patients with transient focal neurological symptoms suggestive of TIA. Patients were classified according to the Precise Diagnostic Score (PREDISC), and early stroke risk was assessed using the ABCD2 score. Serum GPVI levels were measured using enzyme-linked immunosorbent assay. Patients were followed for cerebrovascular events at 2 and 7 days. Associations between GPVI levels, clinical scores, and early outcomes were analyzed, and diagnostic performance was assessed using receiver operating characteristic analysis. **Results:** Serum GPVI levels differed significantly across PREDISC categories and increased from the “TIA unlikely” group to the “TIA very likely” group (*p* < 0.001). GPVI levels were also higher in patients with ABCD2 scores ≥4 than in those with lower scores (*p* < 0.001). GPVI showed positive correlations with both PREDISCs (Spearman’s r = 0.682, *p* = 0.001) and ABCD2 scores (Spearman’s r = 0.469, *p* = 0.001). All early cerebrovascular events occurred in the high-risk ABCD2 group. Patients who experienced cerebrovascular events had higher baseline GPVI levels, with a small-to-moderate effect size for 2-day outcomes (r = 0.22) and a moderate effect size for 7-day outcomes (r = 0.36). ROC analysis demonstrated discriminative performance for identifying patients classified as “TIA very likely” according to the PREDISC system, with an area under the curve of 0.821 (95% CI: 0.71–0.91). **Conclusions:** Serum GPVI levels were associated with PREDISC-based clinical TIA likelihood, higher ABCD2-defined risk categories, and early cerebrovascular events in patients with suspected TIA. These findings suggest that GPVI may have a potential complementary role within structured emergency department–based diagnostic assessment and short-term risk stratification frameworks, pending validation in larger independent cohorts. However, because of the exploratory single-center design, modest sample size, limited number of outcome events, and absence of external validation, these findings should be interpreted as hypothesis-generating and require confirmation in larger multicenter studies.

## 1. Introduction

Transient ischemic attack (TIA) is a neurological emergency characterized by transient focal neurological dysfunction without evidence of acute infarction. Despite its transient nature, TIA carries a substantial early risk of ischemic stroke, with approximately 15% of patients developing stroke within three months, nearly half occurring within the first 48 h [1]. Therefore, early recognition and appropriate risk stratification are essential, and TIA should be considered alongside ischemic stroke in acute clinical settings [2].

However, TIA diagnosis remains challenging due to the lack of a definitive diagnostic test. Clinicians primarily rely on patient history and physical examination, which may lead to diagnostic uncertainty and interobserver variability. To address this limitation, clinical scoring systems and biomarkers have been developed [3].

The ABCD2 score is widely used to estimate short-term stroke risk, while the Precise Diagnostic Score (PREDISC) aims to improve diagnostic classification based on clinical and imaging findings [4,5]. Nevertheless, these tools have inherent limitations, including reliance on subjective clinical assessment, highlighting the need for objective biomarkers.

Platelet activation plays a central role in thromboembolic events. Glycoprotein VI (GPVI), a key platelet collagen receptor, mediates platelet adhesion and activation and has been implicated in arterial thrombosis, including myocardial infarction and ischemic stroke [6]. Targeting GPVI has also been proposed as a potential antithrombotic strategy without increasing bleeding risk [7,8,9].

Despite this biological relevance, the diagnostic and prognostic role of GPVI in suspected TIA has not been clearly established, and its relationship with established clinical risk scores such as ABCD2 remains unclear. Therefore, we aimed to investigate the association of serum GPVI levels with structured diagnostic assessment and short-term risk stratification parameters in patients presenting with suspected TIA in the emergency department. We further investigated the association between GPVI levels, PREDISC classification, and ABCD2 scores and assessed the ability of GPVI to predict early cerebrovascular events.

## 2. Materials and Methods

### 2.1. Study Design and Setting

This prospective, single-center observational study was conducted in the adult emergency department of a tertiary university hospital between 1 April 2024 and 30 May 2024. The study was designed in accordance with the Strengthening the Reporting of Observational Studies in Epidemiology (STROBE) guidelines.

Ethical approval was obtained from the institutional clinical research ethics committee (Approval No: 2024/46, Date: 18 March 2024), and the study was conducted in accordance with the Declaration of Helsinki.

### 2.2. Study Population and Recruitment

Adult patients (≥18 years) presenting to the emergency department with transient focal neurological symptoms suggestive of TIA (e.g., unilateral weakness, sensory deficits, speech disturbance, or transient visual loss) were consecutively screened during the study period.

Patients were evaluated by an emergency physician upon admission. Eligible patients were informed about the study, and written informed consent was obtained prior to inclusion.

### 2.3. Inclusion and Exclusion Criteria

Inclusion criteria were:

- Age ≥ 18 years;
- Presentation with transient neurological symptoms suggestive of TIA;
- Resolution of symptoms at the time of evaluation or during ED observation.

Exclusion criteria were:

- Evidence of intracranial hemorrhage or acute ischemic stroke on initial imaging;
- Persistent neurological deficit;
- Alternative diagnoses explaining symptoms (e.g., seizure, hypoglycemia);
- Refusal to participate.

### 2.4. Clinical Assessment and Data Collection

Upon admission, vital signs including systolic and diastolic blood pressure, heart rate, respiratory rate, oxygen saturation, body temperature, and capillary blood glucose levels were recorded. A detailed medical history, including comorbidities and medication use, was obtained, and a comprehensive physical examination was performed. Information regarding preadmission antiplatelet and anticoagulant therapies, including aspirin, clopidogrel, ticagrelor, and oral anticoagulants, was systematically recorded during the initial clinical assessment. Electrocardiography (ECG) and routine laboratory tests were conducted for all patients. Neuroimaging included brain computed tomography (CT) and diffusion-weighted magnetic resonance imaging (MRI). MRI was performed using a 1.5 Tesla system (GE Revolution Signa, GE Healthcare, Chicago, IL, USA), and all images were evaluated by radiologists blinded to the study.

All patients underwent systematic neurological assessment by both emergency physicians and neurologists during the emergency department evaluation process. Potential TIA-mimicking conditions, including seizure, hypoglycemia, peripheral vertigo, migraine-related neurological symptoms, metabolic disturbances, and functional neurological disorders, were excluded through standardized clinical evaluation, laboratory testing, and neuroimaging findings. Final diagnostic classification was established after integration of clinical presentation, neurological examination findings, diffusion MRI results, and structured PREDISC-based assessment.

### 2.5. Diagnostic Classification and Risk Stratification

All patients were assessed using the Precise Diagnostic Score (PREDISC) and categorized as: 0–1: TIA unlikely, 2–3: TIA possible, 4–8: TIA very likely. The ABCD2 score was calculated based on age, blood pressure, clinical features, duration of symptoms, and diabetes status, with scores ranging from 0 to 7. For outcome analyses and comparative assessment of serum GPVI levels, patients were stratified into two predefined risk categories: Low risk: ABCD2 < 4, High risk: ABCD2 ≥ 4.

TIA diagnostic classification was performed using the Precise Diagnostic Score (PREDISC), a structured clinical and neuroimaging-based classification system developed to improve diagnostic standardization and inter-rater agreement in patients with suspected TIA [4]. The PREDISC system incorporates clinical presentation, diffusion-weighted magnetic resonance imaging (DWI-MRI) findings, and radiological features suggestive of cerebral ischemia.

All patients underwent standardized evaluation by both emergency physicians and neurologists. Neuroimaging findings were interpreted by radiologists who were not involved in the study procedures. Clinical classification according to PREDISC was completed before serum GPVI measurements were analyzed. Investigators performing GPVI measurements were blinded to the clinical classification and follow-up outcomes. Because no universally accepted independent gold-standard diagnostic test exists for TIA, the structured PREDISC-based diagnostic classification was used as the reference standard in this study. In ROC analyses, patients classified as “TIA very likely” (PREDISC 4–8) were considered the high-likelihood TIA group and were compared with the remaining study population.

### 2.6. Follow-Up and Outcome Assessment

Patients were followed up via telephone on day 2 and day 7 after the emergency department visit. The occurrence of cerebrovascular events (stroke or recurrent TIA) during this period was recorded.

### 2.7. Measurement of Serum GPVI Levels

Venous blood samples were collected in standard biochemistry tubes. After clotting, samples were centrifuged at 1000× *g* for 20 min, and serum was separated and stored at −80 °C until analysis.

Serum GPVI levels were measured using a commercially available enzyme-linked immunosorbent assay (ELISA) kit (ELK Biotechnology, Wuhan, China). The assay was performed according to the manufacturer’s instructions.

Optical density was measured at 450 nm using a microplate reader (Multiskan™ GO, Thermo Fisher Scientific, Vantaa, Finland), and concentrations were calculated using a standard calibration curve.

### 2.8. Sample Size and Power Considerations

This study was designed as a prospective pilot study with an exploratory and hypothesis-generating framework aiming to investigate the diagnostic and prognostic significance of serum GPVI levels in patients with suspected transient ischemic attack. Because of the limited availability of prior data regarding serum GPVI levels in patients with suspected transient ischemic attack during the study planning phase, a formal a priori sample size calculation could not be performed. However, following study completion, a post hoc power analysis was conducted based on the primary ROC analysis evaluating the discriminative performance of serum GPVI levels for identifying patients classified as “TIA very likely” according to the PREDISC system. Using the observed discriminative performance from the primary ROC analysis (AUC = 0.821, compared against a null hypothesis AUC of 0.5), the available sample size was estimated to provide greater than 80% statistical power at a significance level of α = 0.05. Statistical power analysis was performed using G*Power software version 3.1 (Heinrich Heine University, Düsseldorf, Germany) [10]. Given the exploratory nature of the study, the findings were intended to be interpreted as hypothesis-generating rather than confirmatory.

### 2.9. Statistical Analyses

Statistical analyses were performed using SPSS for Mac version 26. The normality of the data was assessed with the Shapiro–Wilk test. Descriptive statistics for normally distributed variables are presented as the means and standard deviations (SDs). For comparisons of continuous variables with a normal distribution, the independent-samples Student’s *t* test was used for two-group comparisons, and one-way ANOVA was used for three-group comparisons (with Bonferroni post hoc analysis). For nonnormally distributed data, the Mann–Whitney U test (two groups) or the Kruskal–Wallis test (three groups) was used. Post hoc pairwise comparisons were analyzed with the Kruskal–Wallis test and the Mann–Whitney U test. Correlations between nonnormally distributed variables were evaluated using Spearman’s correlation test. The diagnostic performance of the variables in predicting TIA was assessed by ROC curve analysis. When a meaningful cutoff value was identified, the sensitivity and specificity for that cutoff were reported. An area under the curve with a type I error <0.05 was considered statistically significant. Categorical data are summarized in cross-tabulations and were compared using the chi-square test or Fisher’s exact test, as appropriate. A *p*-value < 0.05 was considered to indicate statistical significance. Because of the limited number of short-term cerebrovascular outcome events, adjusted multivariable regression analyses for 2-day and 7-day outcomes were not performed. Specifically, only four events occurred within 2 days, and seven events occurred within 7 days, which would have created a substantial risk of model overfitting and unstable effect estimates. Therefore, the prognostic analyses were restricted to unadjusted comparisons and effect size reporting, and these findings were interpreted as exploratory.

## 3. Results

### 3.1. Baseline Characteristics of the Study Population

Baseline clinical and demographic characteristics of the study population were evaluated before the analysis of serum GPVI levels. A total of 85 patients with suspected transient ischemic attacks were included. Of these, 38 patients were female and 47 were male. The mean age was 64.5 ± 12.7 years, with a median age of 66 years and an age range of 27–86 years.

Comorbid conditions were present in 68.2% of patients. Hypertension was the most frequent comorbidity, followed by diabetes mellitus. Other recorded comorbidities included coronary artery disease, hyperlipidemia, atrial fibrillation, and chronic kidney disease. Preadmission antiplatelet and anticoagulant therapies were also recorded because of their potential influence on platelet activation and GPVI expression. On electrocardiographic evaluation, most patients were in sinus rhythm, whereas atrial fibrillation was detected in 15.3% of patients. Diffusion-weighted brain MRI revealed acute ischemic lesions in 29.4% of patients. Descriptive data for serum GPVI levels, PREDISC classification, ABCD2 risk categories, and baseline antithrombotic therapy use are presented in Table 1.

### 3.2. GPVI Levels According to PREDISC Classification

**Figure 1:**
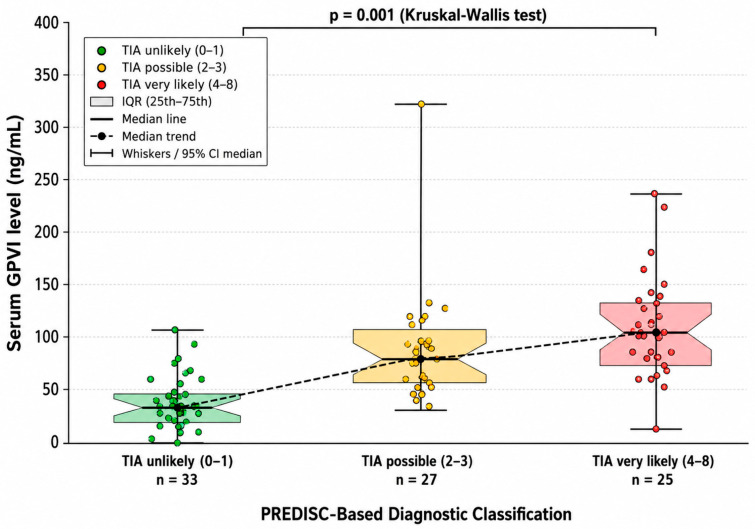
Distribution of serum GPVI levels according to PREDISC-based diagnostic classification categories. Box-and-whisker plots with individual jittered data points demonstrate the distribution of serum GPVI levels across the “TIA unlikely,” “TIA possible,” and “TIA very likely” groups. Boxes represent the interquartile range, horizontal lines within boxes indicate median values, and whiskers/error bars represent the 95% confidence interval of the median. Serum GPVI levels increased progressively across PREDISC categories. GPVI, glycoprotein VI; PREDISC, Precise Diagnostic Score; TIA, transient ischemic attack.

When patients were grouped according to the PREDISC, GPVI levels differed significantly across the three categories (*p* = 0.001). Post hoc analysis demonstrated that GPVI levels were lowest in the “TIA unlikely” group and highest in the “TIA very likely” group (“TIA very likely” > “TIA possible” > “TIA unlikely”; Table 2). Serum GPVI levels differed significantly across PREDISC-based diagnostic classification categories and showed a progressive increase from the “TIA unlikely” group to the “TIA very likely” group (Figure 1). No other variables differed significantly between PREDISC groups (*p* > 0.05).

### 3.3. Patient Stratification According to the ABCD2 Risk Score

All ABCD2-based analyses were performed using the entire study cohort (n = 85), including patients from all PREDISC diagnostic categories. To further evaluate clinical risk stratification, patients were categorized according to their ABCD2 scores. A significant difference was observed in diffusion MRI findings between risk groups: 81.5% of patients with diffusion-positive lesions were classified in the high-risk group (ABCD2 ≥ 4), whereas 18.5% were in the low-risk group (ABCD2 < 4) (*p* = 0.001; Table 3). No significant differences were observed in other baseline characteristics between groups (*p* > 0.05). These results demonstrate that higher ABCD2 scores are associated with objective ischemic findings, reinforcing the validity of clinical risk stratification.

### 3.4. Association Between GPVI Levels and ABCD2 Score

**Figure 2:**
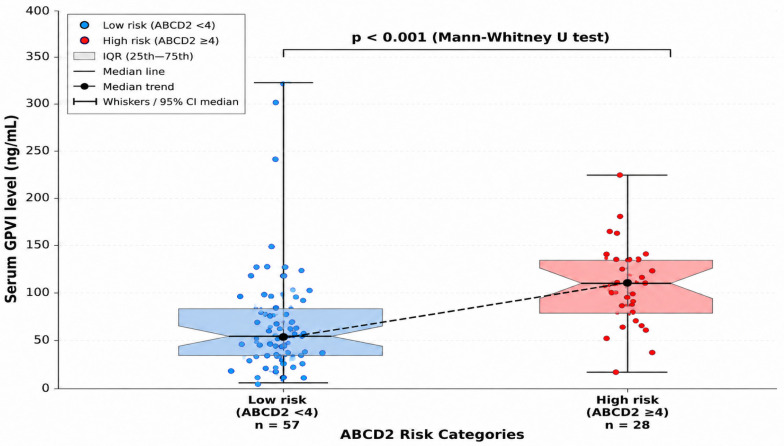
Distribution of serum GPVI levels according to ABCD2 risk categories. Box-and-whisker plots with individual jittered data points demonstrate the distribution of serum GPVI levels in the low-risk (ABCD2 < 4) and high-risk (ABCD2 ≥ 4) groups. Boxes represent the interquartile range, horizontal lines within boxes indicate median values, and whiskers/error bars represent the 95% confidence interval of the median. GPVI, glycoprotein VI; ABCD2, age, blood pressure, clinical features, duration of symptoms, and diabetes score.

Serum GPVI levels differed significantly between ABCD2 risk groups (*p* < 0.001), with higher GPVI levels observed in the high-risk group (ABCD2 ≥ 4) compared with the low-risk group (ABCD2 < 4) (Figure 2, Table 3). Significant differences were also observed between ABCD2 risk groups with respect to age, systolic blood pressure, hypertension frequency, and diffusion MRI findings (Table 3).

### 3.5. Correlation Analyses

Significant positive correlations were observed between serum GPVI levels and both PREDISCs (Spearman’s r = 0.682, *p* = 0.001) and ABCD2 scores (r = 0.469, *p* = 0.001). These findings indicate that higher GPVI levels were associated with increasing clinical TIA likelihood and higher short-term cerebrovascular risk scores.

### 3.6. Diagnostic Performance of GPVI (ROC Analysis)

**Figure 3:**
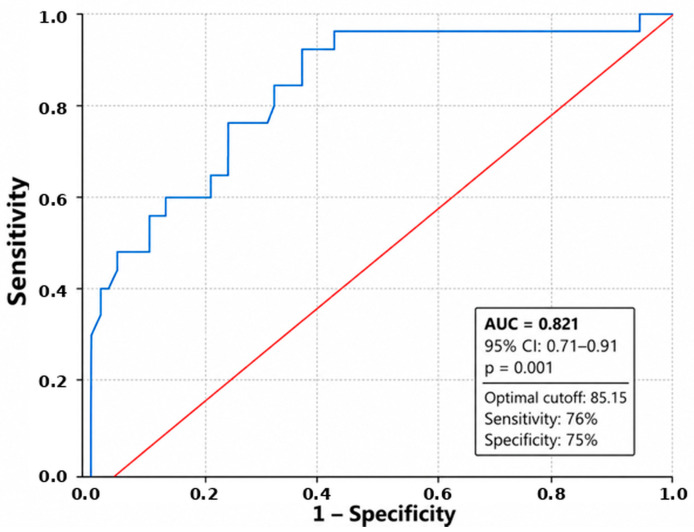
ROC analysis of serum GPVI levels for discrimination of patients classified as “TIA very likely” according to the PREDISC system. The area under the curve was 0.821 (95% CI: 0.71–0.91; *p* = 0.001). The optimal cutoff value was 85.15 ng/mL, with 76% sensitivity and 75% specificity. The blue line represents the ROC curve for serum GPVI, whereas the red diagonal line represents the reference line of no discrimination (AUC = 0.5). GPVI, glycoprotein VI; ROC, receiver operating characteristic; AUC, area under the curve; CI, confidence interval; PREDISC, Precise Diagnostic Score; TIA, transient ischemic attack.

ROC curve analysis was performed using the PREDISC-based diagnostic classification as the reference standard. Patients categorized as “TIA very likely” (PREDISC 4–8) were compared with the remaining study population. Serum GPVI levels demonstrated good discriminative performance for identifying patients with a high clinical likelihood of TIA according to the PREDISC classification system, with an area under the curve (AUC) of 0.821 (95% CI: 0.71–0.91; *p* = 0.001). The optimal cutoff value was 85.15, yielding 76% sensitivity and 75% specificity (Figure 3).

### 3.7. Short-Term Outcomes and Prognostic Value of GPVI

All cerebrovascular events observed during follow-up occurred in patients with an ABCD2 score ≥4. Specifically, all patients who experienced a stroke within 2 days (n = 4) or within 7 days (n = 7) were in the high-risk group (*p* = 0.003 and *p* = 0.001, respectively; Table 4).

Patients who experienced stroke had significantly higher baseline GPVI levels compared with those who remained event-free (Table 5). The difference remained statistically significant for both the 2-day (*p* = 0.046) and 7-day outcome analyses (*p* = 0.001). Effect size analyses demonstrated a small-to-moderate association for the 2-day analysis (r = 0.22) and a moderate association for the 7-day analysis (r = 0.36). However, these findings should be interpreted cautiously because of the limited number of outcome events.

### 3.8. GPVI Levels According to Symptom Duration

When patients were stratified by symptom duration (≤10 min, 11–59 min, ≥60 min), GPVI levels differed significantly among groups (*p* = 0.015). Post hoc analysis revealed that patients with the shortest symptom duration (≤10 min) had lower GPVI levels compared to the other groups (Table 6). This suggests that GPVI levels may also be associated with symptom duration and clinical severity.

Overall, these findings suggest that serum GPVI may be associated with both structured diagnostic classification and early cerebrovascular risk in patients with suspected TIA. However, this exploratory observation requires confirmation in larger prospective cohorts.

## 4. Discussion

In this study, we evaluated the association of serum GPVI levels with PREDISC-based diagnostic classification and ABCD2-defined short-term cerebrovascular risk profiles in patients presenting with suspected TIA in the emergency department. By stratifying patients according to the PREDISC and analyzing their ABCD2 risk profiles, we demonstrated that GPVI levels are associated with both the clinical likelihood of TIA and short-term cerebrovascular risk. Specifically, GPVI levels increased in parallel with PREDISC categories, were higher in patients with ABCD2 scores ≥4, and were significantly elevated in patients who experienced early cerebrovascular events. These findings suggest that GPVI may represent a candidate complementary biomarker within structured diagnostic classification and short-term cerebrovascular risk stratification frameworks in patients with suspected TIA, although external validation is required before any clinical applicability can be established.

Patients who experience a TIA face a substantial risk of subsequent stroke, which is associated with high morbidity and mortality [1,3]. Therefore, accurate diagnosis of suspected TIA cases in the emergency department is essential for timely management and secondary prevention. Early identification and appropriate intervention can significantly reduce the risk of future cerebrovascular events.

GPVI is a key platelet surface receptor that regulates platelet–collagen interactions and plays a central role in thrombus formation. Previous studies have demonstrated that GPVI levels increase in TIA and other ischemic conditions, suggesting its potential as a biomarker [11,12,13]. Elevated GPVI levels reflect enhanced platelet activation and a prothrombotic state, providing a biologically plausible mechanism linking GPVI to cerebrovascular events. Consistent with these observations, our findings suggest that elevated GPVI levels may reflect broader platelet-mediated thromboinflammatory activity associated with both structured diagnostic classification and short-term cerebrovascular risk profiles in patients with suspected TIA.

Despite advances in clinical assessment, TIA diagnosis remains largely dependent on patient history and clinical evaluation. In this context, scoring systems such as PREDISC aim to standardize diagnostic approaches by incorporating clinical and imaging findings [4]. However, the lack of objective biomarkers continues to limit diagnostic accuracy. In our study, GPVI levels differed significantly across PREDISC-defined categories and showed a strong positive correlation with diagnostic probability. These findings suggest that GPVI may represent a biologically relevant marker associated with structured diagnostic classification in patients with suspected TIA.

Experimental and translational studies have highlighted GPVI-mediated platelet signaling pathways as important contributors to thromboinflammatory responses and thrombus formation in ischemic cerebrovascular disease [6,7,8,9,14]. In addition, previous clinical studies have demonstrated increased GPVI expression and elevated soluble GPVI levels in patients with ischemic stroke [11,12,13]. Together, these observations support the biological plausibility of GPVI involvement in cerebrovascular ischemic events and provide mechanistic support for the associations observed in the present study.

Another key finding of this study is the relationship between GPVI levels and the ABCD2 score. The ABCD2 score is a well-established tool for predicting early stroke risk after TIA and is widely used in clinical practice [15]. Multiple studies have confirmed its predictive value and clinical utility in emergency settings [16,17,18]. In our study, GPVI levels were significantly higher in patients with ABCD2 scores ≥4, and all early cerebrovascular events occurred within this high-risk group. These findings suggest that higher GPVI levels may be associated with established short-term cerebrovascular risk categories.

Importantly, the ABCD2 score was originally developed for prediction of early stroke risk following clinically diagnosed TIA rather than for differentiation of TIA from TIA-mimicking conditions. Therefore, ABCD2 was not used as a diagnostic classification tool in the present study but rather as a predefined clinical risk stratification instrument within a cohort of patients with suspected TIA. Nevertheless, because patients with higher ABCD2 scores may also have a higher likelihood of true ischemic events, the observed association between GPVI levels and ABCD2 categories may partly reflect overlap between prognostic risk stratification and structured diagnostic classification. This potential diagnostic enrichment effect should be considered when interpreting the relationship between GPVI and ABCD2-based risk groups.

Notably, to our knowledge, no previous study has specifically examined the relationship between GPVI levels and the ABCD2 score. Our findings suggest that the combination of a clinical risk score and a biomarker may provide complementary information regarding cerebrovascular risk profiles. Elevated GPVI levels may reflect an underlying prothrombotic state, identifying patients who are particularly vulnerable to early cerebrovascular events. Such observations may indicate a possible association between elevated GPVI levels and higher-risk clinical profiles, although this requires confirmation in larger prospective studies.

The association between GPVI levels and structured PREDISC-based diagnostic classification was further explored using ROC analysis, which demonstrated good discriminative ability for identifying patients classified as having a high clinical likelihood of TIA according to the PREDISC system (AUC = 0.821). The identified cutoff value demonstrated moderate discriminative performance within the context of the structured PREDISC-based classification framework. The cutoff value derived from the ROC analysis should be regarded as an internally derived exploratory threshold and should not be interpreted as a clinically applicable decision cutoff or generalized beyond this cohort without independent external validation. Previous studies have reported different GPVI thresholds in stroke populations, likely reflecting variations in assay methodology, patient characteristics, and diagnostic classification strategies [12,13]. These discrepancies highlight the need for standardization in biomarker measurement and validation across independent cohorts. Importantly, because no universally accepted gold-standard diagnostic test exists for TIA, our findings should be interpreted within the context of a structured clinical and imaging-based classification framework rather than definitive TIA diagnosis. Nevertheless, these findings support further investigation of GPVI as a complementary biomarker within structured clinical and imaging-based TIA classification approaches.

Overall, our findings suggest that serum GPVI levels are associated with structured clinical and imaging-based assessment frameworks in patients with suspected TIA; however, its clinical applicability requires validation in larger, externally validated multicenter cohorts.

### Study Limitations

This study has several limitations. First, the relatively small sample size and single-center design may limit the generalizability of the findings. In addition, the prospective observational cohort design and relatively short follow-up period preclude definitive conclusions regarding causal relationships between GPVI levels and cerebrovascular outcomes. Another important limitation is the absence of a universally accepted independent gold-standard diagnostic test for TIA. Therefore, diagnostic classification was performed using the structured PREDISC system, which integrates clinical and imaging-based assessment. Although PREDISC is a standardized tool developed to improve diagnostic consistency and inter-rater agreement, the possibility of diagnostic misclassification cannot be completely excluded.

Potential confounding factors affecting GPVI levels, including medications and comorbid conditions, were also not fully controlled. In particular, preadmission antiplatelet and anticoagulant therapies may have influenced platelet activation status and circulating GPVI levels. Therefore, some of the observed GPVI differences may partly reflect variations in medication exposure rather than exclusively disease-related thromboinflammatory mechanisms. Future studies incorporating medication-adjusted analyses and standardized antithrombotic treatment stratification are warranted to better clarify the independent relationship between GPVI levels and TIA-related cerebrovascular risk.

The use of diffusion-weighted MRI without additional perfusion imaging may also have contributed to underdetection of ischemic changes. Furthermore, variability in GPVI assay techniques may affect comparability across studies.

Another important limitation was the low number of short-term cerebrovascular outcome events observed during follow-up. Because the 2-day and 7-day outcome analyses were based on a limited number of stroke events, the associated *p*-values and effect estimates should be interpreted cautiously. In addition, adjusted multivariable regression analyses were not performed because the small number of outcome events would have increased the risk of model overfitting and unstable estimates. Therefore, the short-term prognostic analyses were restricted to unadjusted comparisons and effect size reporting. Given the exploratory and pilot nature of the study, the findings should be considered hypothesis-generating and require validation in larger independent multicenter cohorts using standardized diagnostic and biomarker assessment protocols and adequately powered multivariable analyses.

## 5. Conclusions

In conclusion, elevated serum GPVI levels were associated with structured PREDISC-based diagnostic classification and with clinical profiles characterized by higher ABCD2-defined cerebrovascular risk in patients with suspected TIA. These findings support the biological and exploratory clinical relevance of GPVI within structured diagnostic and short-term risk assessment frameworks; however, given the pilot and hypothesis-generating nature of the study, the findings should be interpreted cautiously until validated in larger multicenter cohorts.

## Figures and Tables

**Table 1:** Distribution of serum GPVI levels, diagnostic classifications, ABCD2 risk categories, and baseline antithrombotic medication use in the study population.

Variable	n	Mean	SD	Median	Min	Max
GPVI level	85	79.79	54.18	69.3	2.6	324.3
PREDISC	85	3.04	2.67	3	0	8
ABCD2 score	85	3.06	1.643	3	1	7
PREDISC			n	%		
	Unlikely TIA		33	38.8		
	Possible TIA		27	31.8		
	Very likely TIA		25	29.4		
		Total	85	100		
ABCD2 score		<4	57	67.1		
		≥4	28	32.9		
		Total	85	100		
Antiplatelet therapy		No	67	78.8		
		Yes	18	21.2		
Anticoagulant therapy		No	72	84.7		
		Yes	13	15.3		

Mean = arithmetic mean; SD = standard deviation; Min = minimum; Max = maximum; GPVI = glycoprotein VI; PREDISC = Precise Diagnostic Score; TIA = transient ischemic attack. Antiplatelet therapy included aspirin, clopidogrel, or ticagrelor use. Anticoagulant therapy included oral anticoagulant treatment.

**Table 2:** Patient Characteristics and GPVI Levels by PREDISC Category.

*Distribution of Categorical Variables by PREDISC Category*								
		**TIA Unlikely**		**TIA Possible**		**TIA Very Likely**		*p* *
Sex								
Female		18 (54.50%)		12 (44.50%)		11 (44.00%)		0.127
Male		15 (45.50%)		15 (55.50%)		14 (56.00%)		
Comorbidity								
No		14 (42.40%)		5 (18.50%)		8 (32.00%)		0.141
Yes		19 (57.60%)		22 (81.50%)		17 (68.00%)		
Diabetes								
No		25 (75.80%)		16 (59.30%)		15 (60.00%)		0.311
Yes		8 (24.20%)		11 (40.70%)		10 (40.00%)		
Hypertension								
No		16 (48.50%)		8 (29.60%)		12 (48.00%)		0.269
Yes		17 (51.50%)		19 (70.40%)		13 (52.00%)		
Coronary artery disease								
No		24 (72.70%)		21 (77.80%)		22 (88.00%)		0.369
Yes		9 (27.30%)		6 (22.20%)		3 (12.00%)		
ECG rhythm								
SR		31 (93.90%)		20 (74.10%)		21 (84.00%)		0.103
AF		2 (6.10%)		7 (25.90%)		4 (16.00%)		
*Comparison of GPVI levels, ages and vital signs of patients stratified by PREDISC category*								
	**No.**		**N**	**Mean**	**SD**	**Median**	***p***	**Post Hoc**
GPVI (ng/mL)	1	TIA unlikely	33	41.04	25.11	37.6	0.001 **	3 > 2 > 1
	2	TIA possible	27	92.77	54.68	80.1		
	3	TIA very likely	25	116.91	50.05	116		
Age (years)	1	TIA unlikely	33	61.64	14.65	62	0.107 **	
	2	TIA possible	27	68.59	10.95	70		
	3	TIA very likely	25	64.04	11.23	66		
SBP (mm/Hg)	1	TIA unlikely	33	136.55	26.06	128	0.138 ***	
	2	TIA possible	27	153.56	28.44	149		
	3	TIA very likely	25	148.12	22.65	143		
DBP (mm/Hg)	1	TIA unlikely	33	77.61	11.76	76	0.056 **	
	2	TIA possible	27	85.22	15.18	88		
	3	TIA very likely	25	76.48	11.00	76		

* Chi-square test: TIA, transient ischemic attack. ** Kruskal–Wallis test, *** one-way ANOVA: GPVI, glycoprotein VI; SBP, systolic blood pressure; DBP, diastolic blood pressure.

**Table 3:** Comparisons of sex, comorbidities, ECG findings, MRI findings, GPVI levels, age, and vital signs of patients stratified by the ABCD2 score.

*Categorical Variables Stratified by the ABCD2 Risk Group*						
	**ABCD2 Score < 4 (n = 57)**		**ABCD2 Score ≥ 4 (n = 28)**			*p* *
Sex						
Female	26 (45.6%)		12 (42.9%)			0.874 *
Male	31 (54.4%)		16 (57.1%)			
Comorbidity						
No	21 (36.8%)		6 (21.4%)			0.331 *
Yes	36 (63.2%)		22 (78.6%)			
Diabetes						
No	41 (71.9%)		15 (53.6%)			0.127 *
Yes	16 (28.1%)		13 (46.4%)			
Hypertension						
No	31 (54.4%)		5 (17.9%)			0.021 *
Yes	26 (45.6%)		23 (82.1%)			
Coronary artery disease						
No	47 (82.5%)		18 (64.3%)			0.064 *
Yes	10 (17.5%)		10 (35.7%)			
ECG rhythm						
SR	51 (89.5%)		21 (75.0%)			0.151 *
AF	6 (10.5%)		7 (25.0%)			
MRI findings						
No	48 (84.2%)		8 (28.6%)			<0.001 *
Yes	9 (15.8%)		20 (71.4%)			
*Comparison of GPVI levels, ages and vital signs of patients stratified by* *ABCD2 score*						
	**ABCD2 Score**	**n**	**Mean**	**SD**	**Median**	***p***
GP-VI (ng/mL)	<4	57	65.92	53.53	54.3	<0.001 **
	≥4	28	108.00	44.25	112.0	
Age	<4	57	61.46	14.08	63	0.011 ***
	≥4	28	70.68	8.03	71	
SBP (mm/Hg)	<4	57	134.89	28.02	132	<0.001 ***
	≥4	28	169.18	24.94	170	
DBP (mm/Hg)	<4	57	76.72	11.43	76	0.064 **
	≥4	28	87.21	14.31	89	

* Chi-square test, ** Mann–Whitney U test, *** Student’s *t* test. GPVI, glycoprotein VI; SBP, systolic blood pressure; DBP, diastolic blood pressure.

**Table 4:** ABCD2 score and the occurrence of stroke (cerebrovascular event, CVE).

Time Frame	Stroke	ABCD2 Score < 4 (n = 57)	ABCD2 Score ≥ 4 (n = 28)	*p*-Value
Within 2 days	No	57 (100%)	24 (85.7%)	0.003
	Yes	0 (0%)	4 (14.3%)	
Within 7 days	No	57 (100%)	21 (75.0%)	0.001
	Yes	0 (0%)	7 (25.0%)	

Chi-square test. CVE: cerebrovascular event (stroke).

**Table 5:** Comparison of the 2-day and 7-day stroke outcomes according to the GPVI levels.

Outcome Time Frame	Stroke	N	Mean GPVI Level	SD	Median	*p*-Value	Effect Size (r)
Within 2 days	No	81	77.97	54.72	66	0.046	0.22
	Yes	4	116.48	21.12	120		
Within 7 days	No	78	74.07	51.51	64	0.001	0.36
	Yes	7	143.51	43.40	137		

Mann–Whitney U test. Effect sizes were calculated using the rank-biserial correlation coefficient (r). GPVI: glycoprotein VI (ng/mL).

**Table 6:** Comparison of the GPVI levels in patients stratified by symptom duration.

Group	Symptom Duration	n	Mean GPVI	SD	Median	*p*-Value	Post Hoc
1	≤10 min	27	63.81	61.19	54.3	0.015	G1 < G2, G3
2	11–59 min	42	71.75	21.82	76.39		
3	≥60 min	16	99.76	57.30	83.85		

Kruskal–Wallis test. GPVI: glycoprotein VI (ng/mL).

## Data Availability

The datasets generated and/or analyzed during the current study are not publicly available due to patient privacy, confidentiality, and institutional ethical restrictions. De-identified data may be made available from the corresponding author upon reasonable request, subject to approval by the relevant institutional ethics committee and applicable data-sharing regulations.
